# Acute Effects of 2C-E in Humans: An Observational Study

**DOI:** 10.3389/fphar.2020.00233

**Published:** 2020-03-18

**Authors:** Esther Papaseit, Eulalia Olesti, Clara Pérez-Mañá, Marta Torrens, Marc Grifell, Mireia Ventura, Oscar J. Pozo, Elizabeth B. de Sousa Fernandes Perna, Johannes G. Ramaekers, Rafael de la Torre, Magí Farré

**Affiliations:** ^1^Clinical Pharmacology Unit, Hospital Universitari Germans Trias i Pujol-Institut de Recerca Germans Trias i Pujol (HUGTiP-IGTP), Badalona, Spain; ^2^Department of Pharmacology, Therapeutics and Toxicology and Department of Psychiatry and Forensic Medicine, Universitat Autònoma de Barcelona (UAB), Barcelona, Spain; ^3^Integrative Pharmacology and Systems Neuroscience Research Group, Neurosciences Research Program, Hospital del Mar Medical Research Institute (IMIM), Barcelona, Spain; ^4^Universitat Pompeu Fabra, Departament de Ciències Experimentals i de la Salut (CEXS-UPF), Barcelona, Spain; ^5^Drug Addiction Program, Institut de Neuropsiquiatria i Adiccions (INAD), Barcelona, Spain; ^6^Energy Control, Associació Benestar i Desenvolupament, Barcelona, Spain; ^7^Department of Neuropsychology and Psychopharmacology, Faculty of Psychology and Neuroscience, Maastricht University, Maastricht, Netherlands

**Keywords:** 2C-E (2, 5-Dimethoxy-4-ethylphenethylamine), novel psychoactive substances (NPS), psychedelic, phenylethylamines, psychostimulants

## Abstract

2,5-Dimethoxy-4-ethylphenethylamine (2C-E) is psychedelic phenylethylamine, with a chemical structure similar to mescaline, used as new psychoactive substance (NPS). It inhibits norepinephrine and serotonin uptake and, more relevant, acts as a partial agonist of the serotonin 2A (5-HT_2__A_), 2B (5-HT_2__B_), and (5-HT_2__C_) receptors. Consumers have reported that 2C-E induces mild-moderate psychedelic effects, but its pharmacology in humans, including pharmacological effects and pharmacokinetics, have not yet studied. To assess the acute effects of 2C-E on physiological and subjective effects and evaluate its pharmacokinetics, an observational study was carried-out. Ten recreational users of psychedelics self-administered a single oral dose of 2C-E (6.5, 8, 10, 15, or 25 mg). Blood pressure and heart rate were evaluated at baseline, 2, 4, and 6 h post-administration. Three rating scales were administered to evaluate subjective effects: a set of Visual Analog Scales (VAS), the 49-item short form version of the Addiction Research Centre Inventory (ARCI), and the Evaluation of the Subjective Effects of Substances with Abuse Potential (VESSPA-SSE) at baseline, 2, 4, and 6 h after self-administration. To assess 2C-E concentrations oral fluid (saliva) was collected during 6 h. 2C-E induced primarily alterations in perceptions, hallucinations, and euphoric-mood. Saliva maximal concentrations were achieved 2 h after self-administration. Administration of oral 2C-E at recreational doses produces a group of psychedelic-like effects such to 2C-B and other serotonin-acting drugs.

## Introduction

Classical psychedelics (serotonergic psychedelics) have traditionally been defined as a class of psychoactive substances that induce in humans a wide range of complex physiological, behavioral and psychological effects through serotonin 5-HT2_A_ receptors stimulation (Nichols, 2016). In the past few years, however, phenethylamine psychedelics have emerged as a class of new psychoactive substances (NPS) able to induce similar effects to those of controlled psychedelic substances (Vollenweider, 2001; Aarde and Taffe, 2017). 2C-compounds (2C-s) are ring-substituted phenylethylamines derived from the modification of the mescaline structure with two methoxy groups on the benzene ring (2nd and 5th positions) (Tracy et al., 2017). Although they are widely considered a family of substances with hallucinogenic/psychedelic and psychostimulant properties, information available on their pharmacology and toxicology in humans is very limited.

2,5-Dimethoxy-4-ethylphenethylamine [2C-E, or 2-(4-ethyl-2,5-dimethoxyphenyl) ethanamine] is colloquially known as “Aquarust,” “Eternity,” “Europe,” and “Hummingbird” (Sutherland et al., 2016). Synthesized in 1977 by Alexander Shulgin it is one of the most potent 2C-compounds (Shulgin and Shulgin, 1990). 2C-E is structurally very closely related to other 2C-s and to other well-studied phenethylamine substitutes such as mescaline and MDMA (ecstasy). It first came out the club scene in the mid-1980s as a quick replacement for MDMA which had been banned in the United States. 2C-E then remerged on the psychedelic scene and lately has been present as part of the NPS phenomenon. In fact, 2C-E has been documented as being contained in pills sold as ecstasy in America and Europe (United Nations Office on Drugs and Crime [UNODC], 2014), and more recently in Colombia and other Latin American countries, where it is considered an NPS due to its new presence on the drug market (Observatorio de Drogas de Colombia [ODC], 2017).

Pharmacologically, 2C-E, in a similar manner to other 2C-compounds, inhibits the uptake of serotonin and norepinephrine by membrane transporters (SERT and NET, respectively), although with very low activity in relation to amphetamine (Nagai et al., 2007; Van Vrancken et al., 2013; Eshleman et al., 2014). 2C-E mainly acts as a partial agonist at the 5-HT2_A_, 5-HT2_B_, and 5HT2_C_ receptors (related to its psychedelic effects) (Rickli et al., 2015). Also it binds mostly at the adrenergic α-2 receptor (Rickli et al., 2015).

Relatively little information is available regarding human 2C-E metabolism. Nevertheless, research has suggested that it follows similar metabolic pathways to 2C-Bwhich are carried out by O-demethylation and N-acetylation (Theobald et al., 2007).

With respect to epidemiological data on 2C consumption, the information available from web-based questionnaires and population-based surveys is particularly infrequent. In a self selected sample from the 2013 Global Drug Survey^1^, including 2,282 participants in the United States, reporting attendance to nightclubs in the previous year, 46.4% described lifetime use of at least one of the 58 NPS assessed (age range 16–60 years). Among the psychedelic phenethylamines, consumption of 2C-compounds was the most commonly reported (21.7%), and 8.55% admitted taking 2C-E (*n* = 195) (Palamar et al., 2016). In the latest Global Drug Survey there are no specific data regarding the prevalence of 2C-E (Global Drug Survey [GDS], 2018).

In Australia, national cross-sectional surveys among regular ecstasy users (*n* = 693, year 2010) and regular psychostimulant users *(n* = 1260, years 2012/2013) reported a 2 and 3% prevalence of 2C-E use in the previous 6 months, respectively (Bruno et al., 2012; Matthews et al., 2016). In 2014, a sample of Australian NPS users (*n* = 800) described a 5.9% use in the previous 6 months (Sutherland et al., 2017).

In a survey done in Spain among 230 research chemical users a 25.7% had taken 2C-E in the previous year. It was the fifth most frequent substance consumed, and rarely used in combination with other psychostimulants or psychedelics (2C-E + MDMA 1.8%, 2C-E + amphetamine 0.9%, 2C-E + mephedrone 0.9%, and 2C-E + psilocybin 0.4%) (González et al., 2013). In a recent study in the United States, including 356,413 respondents to the 2008–2016 National Survey on Drug Use and Health, 0.12% reported lifetime novel psychedelic use. Of these, 30.1, 14.8, and 23.9%, reported lifetime use of 2C-B (2,5-dimethoxy-4-bromophenethylamine), 2C-E and 2C-I (2,5-dimethoxy-4-iodophenethylamine), respectively (Sexton et al., 2019).

The first description of 2C-E effects was published in *PiHKAL: A Chemical Love Story*, which considered the drug to be one of the “magical half-dozen” or more intense psychedelic phenethylamines (Shulgin and Shulgin, 1990). In recent years, 2C-E recreational users have reported its effects as being a combination of hallucinogenic and stimulating ones, like those of ecstasy and LSD. Like other psychedelics drugs and 2C compounds, 2C-E at low doses usually produces stimulant effects and increased auditory, visual and tactile sensations. At moderate doses it leads to mild hallucinations, and at high ones can cause the user to experience unpleasant hallucinations and sympathomimetic effects. In general, effects from 2C-E are reportedly more intense in comparison to 2C-B (Dean et al., 2013).

An average dose of 2C-E ranges from 10 to 20 mg (medium dose 15–25 mg, high dose 25–40 mg) although exceptionally elevated doses up to 100 mg have been reported (Dean et al., 2013)^2^. Recommendations for an initial dose are between 6 and 20 mg depending on the user’s previous experience with similar drugs, whilst 3 mg is considerate a “microdose” which produces intense effects on cognitive processes and well-being without the typical ones on consciousness (Polito and Stevenson, 2019). As with most psychedelics, the effects of 2C-E are long-acting, lasting typically for 6–12 h, depending on the dose and individual.

To date, a dozen cases of acute intoxication (tachycardia, hypertension, agitation, delirium, and hallucinations) have been reported (Van Vrancken et al., 2013; Iwersen-Bergmann et al., 2019) and, although very rare, some deaths have been linked to 2C-E (Topeff et al., 2011; Sacks et al., 2012). Alarmingly, no human research has been conducted with 2C-E in spite of the relatively long history of its recreational use and the recent resurgence of interest in psychedelic drugs. The aim of our study was to evaluate the pharmacological effects and pharmacokinetics of 2C-E in recreational users.

## Materials and Methods

### Participants

Ten healthy subjects were selected (4 females and 6 males). Volunteers were recreative drug users who had experienced a 2C-series compound at least once in a lifetime. Exclusion criteria were a history of any serious medical or psychopathological disorder including substance use disorder (except nicotine), a previous serious adverse reaction with 2C-series, and chronic medicines use.

Participants were recruited by word-of-mouth and snowball sampling through the harm reduction, non-governmental organization, Energy Control (ABD). The study protocol was submitted and approved by the Clinical Research Ethics Committee (CEIC Parc de Salut Mar, Barcelona, Spain, ref. 2016/6700/I). It was conducted according to the Declaration of Helsinki recommendations. All the participants were correctly and fully informed, both orally and in writing, of the purpose, methods and means of the study. All of them indicated their agreement to participate and signed an informed consent prior inclusion. Participants received monetary compensation for their participation.

### Design and Treatments

The design was a non-controlled prospective observational study with minimal intervention in subjects who self-administrated 2C-E orally. Most evaluations and procedures were similar to a previous naturalistic observational study evaluating acute effects of 2C-B (Papaseit et al., 2018). Each participant participated in one session. Treatment consisted of oral self-administration of one 2C-E capsule, that they brought to the testing site themselves, which they had obtained from an unknown source. Although no information was available about the synthesis of the drug, similar capsules tested by Energy Control, a harm reduction organization that provides a Drug Checking Service for users, showed that the capsules contained 2C-E at 95% purity with no toxic adulterants. The 2C-B pill content was previously analyzed by means of gas chromatography associated with mass spectrometry (GC/MS). The method used permits to check for most common drugs of abuse including most of the NPSs and to know the exact purity of 2C-E in the powder to prepare dosing by a precision scale (Papaseit et al., 2018). The dose of 2C-E self-administrated was selected by the participants based presumably on their previous experience. The mean 2C-E dose was 11.95 ± 5.30 mg [1 female ingested 6.5 mg, 1 female 8 mg, 5 males 10 mg, 2 subjects (1 male and 1 female) 15 mg, and 1 female 25 mg]. In order to standardize dosing for statistical analysis and to evaluate dose-response relationship, we grouped doses in two intervals: 6.5–10 and 15–25 mg (taken by 7 and 3 subjects, respectively). All the selected doses were well tolerated.

#### Procedures

Prior to study session, the participants were submitted to a general medical examination and a psychiatric diagnostic examination. They received training with respect to questionnaires and procedures employed in the study. Upon arrival, they were questioned about any event that could affect their participation. They were asked to refrain from any drug use 2 days prior to the session. Participants were not allowed to consume alcohol or beverages containing caffeine the previous 24 h. Sessions took place on two different days (5 participants each day and administration were separated by various minutes among participants) at a private club with ambient music and participants could talk, read, or play table games during the session and interact in exception to the evaluation times. Also, they were instructed not to talk about the effects of the substance during the session. Assessments were performed by at baseline (pre-dose) and 2, 4, and 6 h after 2C-E self-administration. The experiment was conducted from 15:00 to 22:00 h. Urine spot samples were collected prior administration to exclude prior substance drug use (benzodiazepines, barbiturates, morphine, cocaine, amphetamines, methamphetamine, MDMA, marijuana, phencyclidine) with Instant-View, Multipanel 10 Test Drug Screen Alfa Scientific Designs Inc., Poway, CA, United States. Self-administration of 2C-E took place around 16.00 h. The sequence of procedures at each time point of the session was: physiological measures, oral fluid collection, and subjective effects questionnaires. A psychiatry was present during the entire session. Adverse effects were assessed during study session.

#### Physiological Effects

Non-invasive systolic and diastolic blood pressure (SBP and DBP), and heart rate (HR) were determined with an Omron^®^ monitor at baseline and 2, 4, and 6 h after administration. Oral temperature was measured simultaneously.

#### Subjective Effects

Subjective effects of 2C-E were reported at baseline and at 2, 4, and 6 h after self-administration. They were measured using a set of Visual Analog Scales (VAS), the 49-item Addiction Research Centre Inventory (ARCI) short form, and the Evaluation of the Subjective Effects of Substances with Abuse Potential (VESSPA-SSE) questionnaires. VAS (100 mm, from “not at all” to “extremely”) were used to rate intensity; stimulated; high; good effects; liking; content; changes in colors; changes in shapes; changes in lights; hallucinations-seeing of lights or spots; hallucinations-seeing animals, things, insects or people; changes in hearing; hallucinations-hearings of sounds or voices; different body feeling; unreal body feeling; changes in distances; different surroundings; unreal surroundings; confusion; fear; depression or sadness; drowsiness; dizziness; bad effects; headache; nausea; vertigo; breathing difficulty and face flushing (González et al., 2015; Papaseit et al., 2016, 2018).

The ARCI 49-item short form is a validated instrument that includes five subscales related to drug sedation (pentobarbital-chlorpromazine-alcohol group, PCAG), euphoria (morphine-benzedrine group, MBG), dysphoria and somatic symptoms (lysergic acid diethylamide group, LSD), intellectual efficiency and energy (benzedrine group, BG) and d-amphetamine-like effects (A) (Lamas et al., 1994; Papaseit et al., 2016; Martínez-Riera et al., 2019).

The VESSPA-SE is a questionnaire that measures changes in subjective effects caused by different drugs including stimulants and psychedelics and includes six subscales: sedation (S), psychosomatic anxiety (ANX), changes in perception (CP), pleasure and sociability (SOC), activity and energy (ACT), and psychotic symptoms (PS) (González et al., 2015; Papaseit et al., 2016).

#### Oral Fluid Concentrations of 2C-E

To assess 2C-E concentrations in oral fluid (saliva), it was collected with Salivette^®^ tubes at baseline, 2, 4, and 6 h after self-administration. After collection samples were centrifuged and frozen at -20°C until analysis. 2C-E concentrations were analyzed by a modified and validated liquid chromatography–mass spectrometry method LC-MS/MS) (Papaseit et al., 2018).

### Statistical Analysis

For physiological (SBP, DBP, HR, and T) and subjective effects (VAS, ARCI, and VESSPA), differences with respect to baseline were calculated. Maximum effects (E_max_) were determined and the area under the curve of the effects (AUC_0 – 6 h_) were calculated using the trapezoidal rule.

For 2C-E oral fluid concentrations, the maximum concentration (C_max_), the time needed to reach the maximum concentration (T_max_) and the AUC_0 – 6 h_ were determined using the Pharmacokinetic Functions for Microsoft Excel (Joel Usansky, Atul Desai, and Diane Tang-Liu, Department of Pharmacokinetics and Drug Metabolism, Allergan, Irvine, CA, United States).

Although it is remarkably that the participant that selected the lowest dose (6.5 mg) presented higher acute effects and oral fluid concentrations in comparison to others, this subject was included in all the analysis.

A one-way analysis of variance (ANOVA) test including all doses as a factor was used for E_max_ and AUC_0 – 6_. When the dose factor was statistically significant, a *post hoc* analysis for the two defined groups were done using a Student *T*-test (lower dose group: 6.5–10 mg, *n* = 7; higher dose group: 10–25 mg, *n* = 3).

To evaluate the effects along time and to study the effects of the substance in comparison to baseline, a one-way repeated measures ANOVA, with time as factor (baseline, 2, 4, and 6 h), was done to evaluate the time-course of effects (for all doses). When the time condition was statistically significant, a Dunnett multiple comparison *post hoc* test was conducted to compare the different time points with baseline (0–2 h, 0–4 h, 0–6 h).

All statistical tests were conducted using PAWS Statistics version 18 (SPSS Inc., Chicago, IL, United States). A *p* < 0.05 value was considered statistically significant.

## Results

### Participants

**FIGURE 1:**
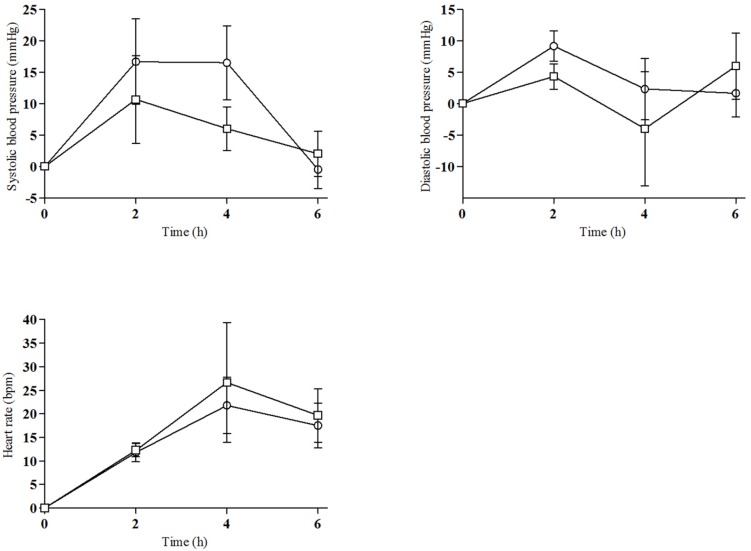
Time course of changes from baseline for physiological effects [°, 6.5–10 mg of 2C-E (*n* = 7), □, 15–25 mg of 2C-E (*n* = 3); mean, standard error].

**FIGURE 2:**
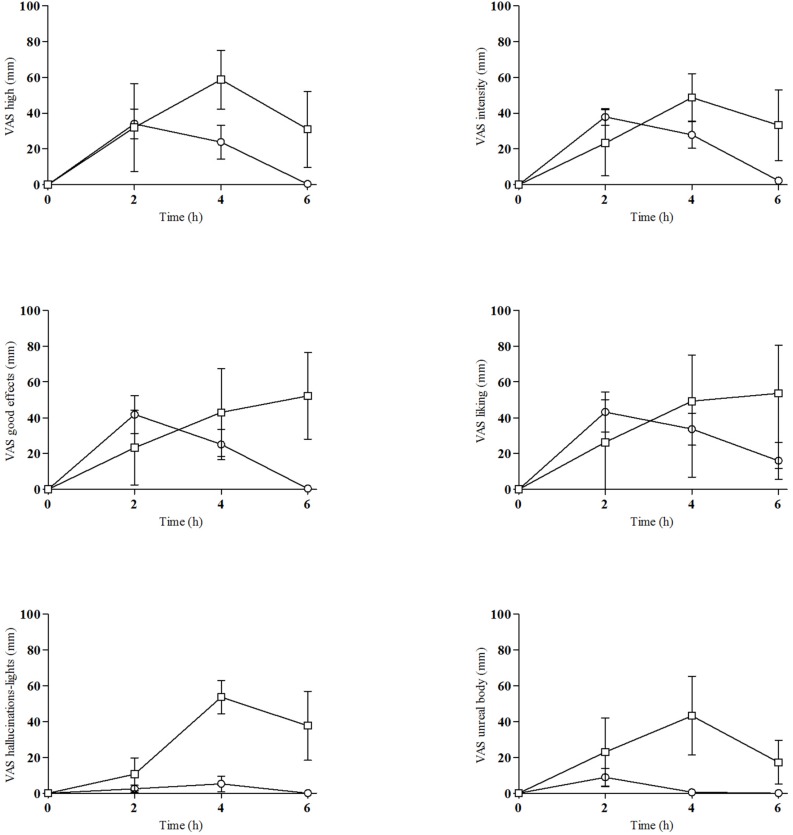
Time course of changes from baseline for subjective effects [°, 6.5–10 mg of 2C-E (*n* = 7); □, 15–25 mg of 2C-E (*n* = 3); mean, standard error].

**FIGURE 3:**
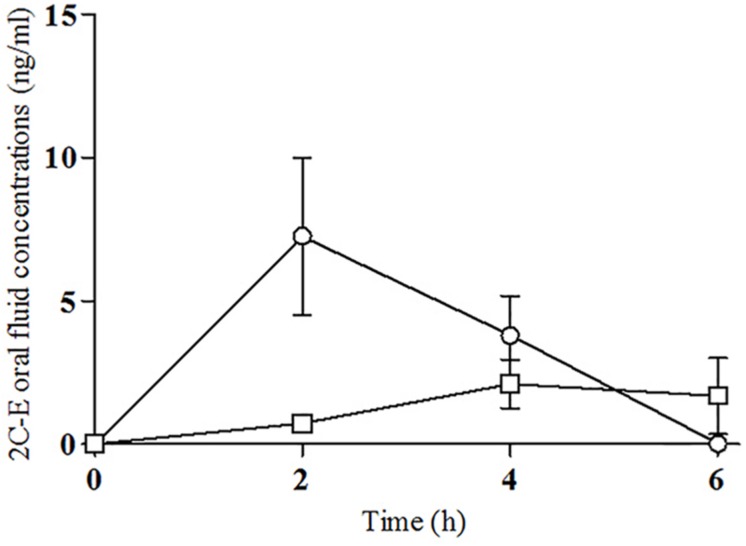
Time course of 2C-E concentrations in oral fluid [Δ, 6.5 mg of 2C-E (*n* = 1); °, 8–10 mg of 2C-E (*n* = 6), □, 15–25 mg of 2C-E (*n* = 3); mean, standard error].

All ten selected subjects participated in the study (4 females and 6 males). Demographics were a mean age of 27 ± 4 years (range 24–37), mean weight of 64.60 ± 8.77 kg (range 58–78), and mean body mass index (BMI) of 20.26 ± 2.55 kg/m^2^ (range 16–24). The mean weight-adjusted dose of 2C-E was 0.19 ± 0.09 mg/kg (range 0.13–0.43). All subjects had previous recreative experience with 2Cs, psychedelics/hallucinogens, cocaine, MDMA, amphetamines, and cannabis. Seven of them were current tobacco smokers (range 0.5–7 cigarettes/day) and all consumed alcohol daily (mean 1.4 units/day). All drugs of abuse urine tests were negative at baseline. As explained in the statistical analysis for dose-response analysis we grouped doses in two groups (6.5, 8–10, and 15–25 mg), Figures 1–3 are showed as the two doses groups. Supplementary Figures S1–S3 presented individual data in order to show the elevated variability of the acute effects and concentrations.

### Physiological Effects

Effects of 2C-E on physiological signs are summarized in Table 1 and Figure 1, and Supplementary Figure S1 (individual data). 2C-E produced a non-significant increase in SBP, DBP, HR and T. For HR significant differences were detected in the comparison of baseline and 4 hand 6 h after administration. Regarding T, only statistically significant differences were detected at 2 and 4 h. No dose-response relationship was observed.

**TABLE 1:** Summary of result on the physiological effects observed after self-administration of 2C-E.

Effects	Parameter	ANOVA			Comparison to baseline	T-Student			
				
		Doses (6.5–25 mg) (*n* = 10)			Doses (6.5–25 mg) (*n* = 10)	6.5–10 mg (*n* = 7)	15–25 mg (*n* = 3)	*T*-value	*p*-value
			
		Mean ± SD	*F*	*p*-value	Dunnett’s test	Mean ± SD	Mean ± SD		
**Physiological effects**									
Systolic blood pressure	E_max_	15 ± 23	0.047	0.995		15 ± 28	15 ± 5.8	ND	ND
	AUC_0__–__6_	41 ± 74	0.050	0.994		43 ± 89	35 ± 22	ND	ND
	T-C				NS				
Diastolic blood pressure	E_max_	1.6 ± 20	0.840	0.554		2 ± 22	0.7 ± 20	ND	ND
	AUC_0__–__6_	−2.1 ± 63	0.873	0.539		−5.9 ± 74	6.7 ± 39	ND	ND
	T-C				NS				
Heart rate	E_max_	18 ± 19	2.883	0.138		12 ± 17	33 ± 19	ND	ND
	AUC_0__–__6_	58 ± 56	4.799	0.058		41 ± 57	98 ± 34	ND	ND
	T-C				b, c				
Temperature	E_max_	0.5 ± 0.2	2.366	0.185		0.1 ± 0.2	0.3 ± 0.2	ND	ND
	AUC_0__–__6_	0.3 ± 0.5	1.122	0.440		0.2 ± 0.5	0.6 ± 0.6	ND	ND
	T-C				b				

**Emax = peak effects 0–6 h (differences from baseline). AUC_0__–__6_ = area under the curve from 0 to 6 h. *Units: mmHg (systolic blood pressure and diastolic blood pressure), beats per minute (heart rate), °C (temperature). For E_*max*_ and AUC_0__–__6_ a one-way ANOVA was used to examine the effect of all doses. A p < 0.05 was considered statistically significant. Only if a statistical difference were detected an unpaired T-Student was used to examine differences between the grouped doses (6.5–10 mg vs. 15–25 mg). A p < 0.05 was considered statistically significant. ND, not done. For T-C a one-way ANOVA and a post hoc Dunnett’s test for multiple comparisons was used. Statistical differences between are presented as “a” p < 0.05, “****a****” p < 0.01 (times 0–2 h), “b” p < 0.05, “****b****” p < 0.01 (times 0–4 h), “c” p < 0.05, “****c****” p < 0.01 (times 0– 6 h).***

### Subjective Effects

The subjective effects induced by 2C-E are presented in Table 2 and Figure 2, and Supplementary Figure S2 (individual data). In summary, 2C-E significantly increased scores for most of the outcomes measured with VAS. Some effects were related to dose, as higher doses produced more intense effects. The substance produced more intensity of effects in comparison to baseline for most variables.

**TABLE 2:** Summary of result on the subjective effects and saliva concentrations observed after self-administration of 2C-E.

Effects	Parameter	ANOVA			Comparison to baseline	T-Student			
				
		Doses (6.5–25 mg) (*n* = 10)			Doses (6.5–25 mg) (*n* = 10)	6.5–10 mg (*n* = 7)	15–25 mg (*n* = 3)	*T*-value	*p*-value
			
		Mean ± SD	*F*	*p*-value	Dunnett’s test	Mean ± SD	Mean ± SD		
**Visual analog scale (VAS)**									
Intensity	E_max_	46±17	1.045	0.468		43±11	55±27	ND	ND
	AUC_0__–__6_	147±68	5.464	0.045		134±52	177±104	−0.916	0.387
	T-C				**a**, **b**				
Stimulated	E_max_	37±28	1.423	0.349		29±25	55±31	ND	ND
	AUC_0__–__6_	114±104	3.666	0.093		86±87	179±130	ND	ND
	T-C				**b**				
High	E_max_	48±23	1.924	0.245		48±21	54±44	ND	ND
	AUC_0__–__6_	145±99	6.003	0.038		134±74	185±189	ND	ND
	T-C				**a**, **b**				
Good effects	E_max_	50±27	0.839	0.555		72±86	62±30	ND	ND
	AUC_0__–__6_	150±110	3.875	0.085		116±74	212±133	ND	ND
	T-C				**a,** b				
Liking	E_max_	51±30	0.751	0.598		49±24	55±48	ND	ND
	AUC_0__–__6_	181±134	1.691	0.287		170±113	205±203	ND	ND
	T-C				**a**, **b**, **c**				
Content	E_max_	47±30	1.048	0.467		44±25	53±47	ND	ND
	AUC_0__–__6_	145±110	1.784	0.269		13092	180±161	ND	ND
	T-C				a, b				
Changes in colors	E_max_	32±21	6.786	0.030		237.9	52±32	−2.426	0.041
	AUC_0__–__6_	102±111	51.871	< 0.001		55±16	209±173	−2.545	0.034
	T-C				**a**, b				
Changes in shapes	E_max_	27±27	3.717	0.091		15±16	53±32	ND	ND
	AUC_0__–__6_	73±91	14.974	0.005		34±35	165±128	−2.665	0.029
	T-C				NS				
Changes in lights	E_max_	35±28	9.468	0.015		23±18	64±32	−2.665	0.029
	AUC_0__–__6_	99±90	34.980	0.001		59±39	193±114	−2.930	0.019
	T-C				**c**				
Hallucinations-seeing of lights or spots	E_max_	21±26	8.564	0.018		6.612	55±16	−5.388	0.001
	AUC_0__–__6_	61±88	13.026	0.007		16±28	16692	−4.220	0.003
	T-C				c				
Hallucinations-seeing animals, things, insects, or people	E_max_	6.2±16	1.002	0.485		1.4±3.8	17±28	ND	ND
	AUC_0__–__6_	11±26	0.987	0.491		2.9±7.6	29±46	ND	ND
	T-C				NS				
Changes in hearing	E_max_	4.1±7.4	15.425	0.005		4.0±8.5	4.3±5.1	−0.062	0.952
	AUC_0__–__6_	12±23	19.891	0.003		12±27	11±14	0.080	0.938
	T-C				NS				
Hallucinations-hearings of sounds or voices	E_max_	2.2±4.9	13.444	0.007		0.0±0.0	7.3±7.0	−3.026	0.016
	AUC_0__–__6_	4.9±11	29.642	0.001		0.0±0.0	16±15	−3.189	0.013
	T-C				NS				
Different body feeling	E_max_	46±23	1.559	0.315		46±20	46±33	ND	ND
	AUC_0__–__6_	135±78	3.792	0.088		120±46	169±133	ND	ND
	T-C				**a**, **b**				
Unreal body feeling	E_max_	20±26	6.413	0.033		9.413	43±38	−2.231	0.056
	AUC_0__–__6_	58±101	26.999	0.001		19±26	150±161	−2.273	0.053
	T-C				NS				
Changes in distances	E_max_	22±30	1.286	0.387		13±25	44±34	ND	ND
	AUC_0__–__6_	60±98	5.499	0.045		26±50	139±149	−1.899	0.094
	T-C				NS				
Different surroundings	E_max_	29±29	2.311	0.191		17±18	56±32	ND	ND
	AUC_0__–__6_	82±100	8.625	0.018		37±38	187±129	−3.001	0.017
	T-C				b, c				
Unreal surroundings	E_max_	13±27	14.432	0.006		0.0±0.0	43±36	−3.428	0.009
	AUC_0__–__6_	45±102	29.938	0.001		0.0±0.0	150±153	−2.843	0.022
	T-C				NS				
Confusion	E_max_	15±22	1.891	0.250		0.0±0.0	2.3±2.08	ND	ND
	AUC_0__–__6_	35±49	6.297	0.034		9±12	30±37	−1.461	0.182
	T-C				NS				
Fear	E_max_	3.1±5.2	0.802	0.573		1.1±3.0	7.7±7.1	ND	ND
	AUC_0__–__6_	6.7±12	0.785	0.581		2.3±6.1	17±16	ND	ND
	T-C				NS				
Depression or sadness	E_max_	3.0±5.3	3.774	0.089		1.3±3.0	7.0±8.2	ND	ND
	AUC_0__–__6_	7.0±12	2.437	0.178		2.6±6.0	17±16	ND	ND
	T-C				NS				
Drowsiness	E_max_	22±28	10.050	0.013		15±18	38±44	−1.221	0.257
	AUC_0__–__6_	66±89	17.533	0.004		48±64	106±140	−0.933	0.378
	T-C				**a**				
Dizziness	E_max_	15±21	1.916	0.246		9.9±16	27±30	ND	ND
	AUC_0__–__6_	44±71	4.783	0.058		22±36	97±114	ND	ND
	T-C				a				
Bad effects	E_max_	8.4±10	2.761	0.147		9.3±12	8.7±4.5	ND	ND
	AUC_0__–__6_	23±29	1.938	0.243		22±33	26±20	ND	ND
	T-C				a				
Headache	E_max_	14±17	1.509	0.327		8.3±12	26±22	ND	ND
	AUC_0__–__6_	28±33	3.647	0.094		25±39	32±22	ND	ND
	T-C				NS				
Nausea	E_max_	11±10	0.262	0.891		11±11	12±7.3	ND	ND
	AUC_0__–__6_	32±30	0.761	0.593		28±31	40±30	ND	ND
	T-C				a				
Vertigo	E_max_	12±20	0.316	0.857		8.7±18	19±26	ND	ND
	AUC_0__–__6_	20±32	0.143	0.959		17±37	25±23	ND	ND
	T-C				NS				
Breathing difficulty	E_max_	2.7±6.5	90.601	< 0.001		0.3±0.8	8.3±11	−2.103	0.069
	AUC_0__–__6_	10±27	319.150	< 0.001		0.6±1.6	32±47	−1.910	0.093
	T-C				NS				
Face flushing	E_max_	13±20	0.374	0.819		16±17	27±29	ND	ND
	AUC_0__–__6_	20±20	0.883	0.535		53±59	72±90	ND	ND
	T-C				NS				
**Addiction research center inventory (ARCI)**									
PCAG (sedation)	E_max_	3.1±4.6	0.443	0.775		3.1±4.3	3.0±6.1	ND	ND
	AUC_0__–__6_	14±13	1.101	0.447		12±13	18±14	ND	ND
	T-C				**a**				
MBG (euphoria)	E_max_	4.4±4.4	0.904	0.526		3.1±3.5	7.3±5.7	ND	ND
	AUC_0__–__6_	16±19	1.549	0.318		11±14	28±28	ND	ND
	T-C				b, **c**				
LSD (dysphoria and somatic symptoms)	E_max_	4.5±2.7	1.469	0.337		3.6±1.0	6.7±2.5	ND	ND
	AUC_0__–__6_	12±9.8	3.802	0.088		7.4±6.3	23±7.55	ND	ND
	T-C				**a**, **b**				
BG (intellectual efficieny and energy)	E_max_	1.5±2.2	0.330	0.847		1.1±2.0	2.3±3.1	ND	ND
	AUC_0__–__6_	4.1±6.6	0.419	0.790		4.0±5.6	4.3±10	ND	ND
	T-C				b				
A (amphetamine-like effects)	E_max_	4.2±1.9	0.755	0.596		3.7±1.2	5.3±2.9	ND	ND
	AUC_0__–__6_	14±8.1	0.658	0.647		13±5.9	19±12	ND	ND
	T-C				**a**, **b**, c				
**Evaluation of subjective effects of substances with abuse potential (VESSPA-SEE)**									
S (sedation)	E_max_	6.7±3.3	9.231	0.016		5.8±3.5	8.7±2.08	−1.275	0.238
	AUC_0__–__6_	19±11	3.051	0.126		16±11	24±12	ND	ND
	T-C				a				
ANX (psychosomatic anxiety)	E_max_	4.0±2.9	1.996	0.234		3.3±3.1	5.7±1.5	ND	ND
	AUC_0__–__6_	13±10	3.178	0.118		11±10	19±8.7	ND	ND
	T-C				a, **b**				
CP (changes in perception)	E_max_	4.2±4.7	8.452	0.019		1.7±1.2	10±4.6	−4.736	0.001
	AUC_0__–__6_	13±17	17.663	0.004		4.33.9	33±18	−4.311	0.003
	T-C				**b**				
SOC (pleasure and sociability)	E_max_	8.2±7.7	2.389	0.183		5.9±5.2	13±11	ND	ND
	AUC_0__–__6_	26±29	3.2±12	0.116		18±20	47±40	ND	ND
	T-C				b				
ACT (activity and energy)	E_max_	6.0±6.3	1.205	0.412		3.9±4.4	11±7.9	ND	ND
	AUC_0__–__6_	18±20	1.362	0.365		11±12	35±27	ND	ND
	T-C				**b**				
PS (psychotic symptoms)	E_max_	3.1±4.1	3.753	0.090		1.2±1.1	7.3±5.7	−2.919	0.019
	AUC_0__–__6_	11±18	15.680	0.005		3.1±3.0	28±17	−2.418	0.042
	T-C				NS				
**Oral fluid concentrations**									
2C-E	C_max_	5.8±6.4	0.491	0.745		7.3±7.2	2.41.7	ND	ND
	AUC_0__–__6_	18±18	0.532	0.720		22±21	7.3±4.7	ND	ND
	T-C				**a**				

**E_*max*_ = peak effects 0–6 h. Emax = peak effects 0–6 h (differences from baseline). AUC_0__–__6_ = area under the curve from 0 to 6 h. *Units: mm [visual analog scale (VAS)], and score [Addiction Research Center Inventory (ARCI), Evaluation of Subjective Effects of Substances with Abuse Potential questionnaire (VESSPA-SEE)] and expressed as mean. C_*max*_ = maximal concentrations 0–6 h (differences from baseline) measured by ng/mL. For Emax and AUC_0__–__6_ a one-way ANOVA was used to examine the effect of all doses. A p < 0.05 was considered statistically significant. Only if a statistical difference were detected an unpaired T-Student was used to examine differences between the grouped doses (6.5–10 mg vs. 15–25 mg). A p < 0.05 was considered statistically significant. ND, not done. For T-C a one-way ANOVA and a post hoc Dunnett’s test for multiple comparisons was used. Statistical differences between are presented as “a” p < 0.05, “**a**” p < 0.01 (times 0–2 h), “b” p < 0.05, “**b**” p < 0.01 (times 0–4 h), “c” p < 0.05, “**c**” p < 0.01 (times 0–6 h).***

For VAS scales related to euphoria-stimulation the highest scores were observed for “intensity,” “stimulated,” “high,” “good effects,” “liking,” and “content.” When compared to baseline, significant differences were detected at 2 and 4 h, except for “stimulated” (4 h) and “liking” (2, 4, and 6 h). No dose-response was observed when comparing both groups of doses.

For VAS scales measuring changes in perceptions, statistically significant differences in E_max_ and AUC_0__–__6_ were detected for all VAS except in “different body feelings.” When compared to baseline, significant differences were found in VAS for “changes in colors” (2 h), “changes in lights” (4 h), “different body feeling” (h, 4 h), and “different surroundings” at 4 h and 6 h. A dose-response was observed in all VAS except for “changes in hearing,” “changes in distances,” and “different body feeling.”

With respect to scales measuring hallucinations, the highest scores were found for “hallucination-seeing of lights/spots” (E_max_ 21.00 mm) whilst modest and low scores were observed for “hallucination-seeing animals, things, insects or people” (E_max_ 6.20 mm, no significant) and “hallucination-hearing of sounds or voices” (E_max_ 2.20 mm, significant). Significant effects, baseline differences and dose-response were observed for “hallucinations-seeing of light and spots” (6 h) and “hallucination-hearing of sounds or voices.”

In addition, 2C-E induced “confusion,” “drowsiness,” and “breathing difficulty.” Differences from baseline were observed for “drowsiness,” “dizziness,” “bad effects,” and “nausea.” No dose-response was observed except for “breathing difficulty.”

In relation to ARCI questionnaire, significant increases in the scores of all subscales were detected, however, differences in dose were not statistically significant. Similarly, differences from baseline were observed for all subscales at different times. No dose-response was observed.

With respect to the VESSPA, significant changes were shown in Sedation, Change in perception and Psychotic symptoms, with significant differences from baseline in all except Psychotic symptoms. Dose-response relationship were detected for Changes in Perception and Psychotic symptoms.

Most of the effects dissipated after 6 h, and all subjects returned to their usual routine. Two of them presented residual mild visual hallucinations (lights) at 6 h which disappeared 1–2 h later.

### Oral Fluid Concentrations

The oral fluid concentration-time curve for 2C-E are shown in Figure 3, and Supplementary Figure S3 (individual data). Concentrations of 2C-E increased rapidly, reaching a peak 2 h after ingestion. Concentrations rapidly decrease from 2 to 6 h after ingestion. Mean maximum concentration (C_max_) values of 5.8 ± 6.4 ng/mL (range 0.93–21.54) were obtained at a T_max_ of 2 h following drug administration. The AUC_0__–__6_ was 18 ± 18 ng⋅h/mL (range 3.69–57.70). Plasma concentrations varied considerably among doses and subjects. No significant differences between the two grouped doses were found for C_max_ or AUC_0__–__6_ (Table 2). All ten subjects presented positive concentrations of 2C-E at 4 h; only 5, however, had 2C-E concentrations in saliva at 6 h.

## Discussion

To the best of our knowledge, this is the first study to assess the acute behavioral (subjective) and physiological effects and oral fluid concentrations of 2C-E after the administration of known doses (6.5–25 mg) in humans. The main finding is that 2C-E induced primarily a group of psychedelic-like effects, a profile consistent with prior data from surveys and poisonings symptoms (Matthews et al., 2016). Moreover, our study provides unique results about concentrations of 2C-E in oral fluid.

In our non-controlled setting, 2C-E only partially mimicked the prototypical sympathomimetic-like effects of other psychedelic and psychostimulant drugs (Schmid et al., 2015; Dolder et al., 2017) and 2C-B (Papaseit et al., 2018). The physiological actions induced by 2C-E included a mild-moderate increase of HR, without changes in blood pressure. The effects were lower than those produced by 2C-B (Papaseit et al., 2018) and by MDMA, mephedrone or other amphetamines administered in dose-controlled conditions (Farré et al., 2015; Papaseit et al., 2016). It is possible that the wide range of doses in the present study (from 6.5 to 25 mg) did not permit differences to be observed in blood pressure when compared to 2C-B (in a narrow range from 10 to 20 mg) (Papaseit et al., 2018). For 2C-E the maximal cardiac effect was observed at the 2 h assessment, maintained over 2–4 h, and returned to baseline at 6 h post-administration.

In this study, 2C-E produced mixed euphoria, pleasure and well-being feelings, and alterations in mental functions like psychedelics such as 2C-B (González et al., 2015; Papaseit et al., 2018), psilocybin (Griffiths et al., 2006), salvinorin A (Johnson et al., 2011) and ayahuasca (Riba et al., 2001, 2004) and psychostimulants such as MDMA (Papaseit et al., 2016), amphetamine (Cami et al., 2000), and mephedrone (Papaseit et al., 2016). Under 2C-E influence participants reported euphoria, stimulation, and altered state of consciousness due to the psychedelic experience. Changes in mood were more pronounced than perceptual ones. As an example, the mean VAS ratings of “high,” “good effects,” and “liking” reached up to 50% of the maximum possible VAS scores, but they were still lower than those observed in experimental dose-controlled conditions for 2C-B, MDMA, and other stimulants as mephedrone (Mas et al., 1999; Farré et al., 2015; Papaseit et al., 2016, 2018). It is possible that euphoria could be an important issue of the psychedelic experience after 2C-B or 2C-E use, as previously postulated for other psychedelics (Bouso et al., 2016). It is noteworthy that 2C-E increased some somatic VAS scales (drowsiness, dizziness, and confusion) in a similar manner to 2C-B.

Moreover, alteration in perception varied from changes in perceptions to hallucinations, that were experienced by 5 volunteers (3 only visual and 2 visual and auditory hallucinations). Of these, 5 subjects reported visual (seeing of lights or spots, 14–72 mm), 1 subject visual (seeing things/people, 50 mm) and 2 participants auditory (hearing sounds/voices, 8–14 mm score), effects. Results differ in intensity from other psychedelics probably because in this study subjects self-administered low to moderate doses of the substance. Additionally, 2C-E produced higher increases in sociability (VESPA SOC subscale) and augmented ratings on change perceptions, effects widely related to MDMA and LSD (Papaseit et al., 2016; Dolder et al., 2017; Puxty et al., 2017). Overall, the subjective effects induced by 2C-E appear to be closely related to psychedelic drugs indicating that it produces mind-altering and hallucinogenic effects which could be primarily mediated by the 5HT_2__A_ receptor.

In a similar manner to 2C-B, the sole 2C-compound with previous observational data in humans after dose-controlled administration, 2C-E induced modest sympathomimetic effects, similar feelings of well-being, euphoria, and changes in perception although with more profound hallucinations (Caudevilla-Gálligo et al., 2012; González et al., 2015; Papaseit et al., 2018).

As expected, in our study 2C-E produced the prototypical effects of psychedelic substances that include visual hallucinations, perceptual changes, somatic symptoms, and activation of euphoria. Although it also induced headache, confusion, and breathing difficulty, no severe adverse reactions were observed. Our results show that in a recreational setting, self-administration of low-moderate doses of 2C-E by healthy experienced users is well tolerated and relatively safe. The results are consistent with a relatively low number of severe acute toxicity cases associated to 2C-E use (Iwersen-Bergmann et al., 2019).

The pharmacokinetics of 2C-E in humans has not yet been fully known. Our results on oral fluid concentrations of 2C-E are the first data in humans to be reported. 2C-E concentrations ranged from 0.93 to 21.54 ng/mL, with an average peak concentration of 5.8 ± 6.4 ng/mL observed at 2 h after administration. Oral fluid 2C-E showed a similar time course with effect outcomes. Nevertheless, because the study included five different 2C-E doses in a limited number of subjects, a dose-concentration relationship was not observed. We do not have an explanation for the high variability observed, with higher concentrations after lower doses. Problems in the collection of the samples or an erratic distribution of 2C-E in saliva could be possible causes. Concentrations in oral fluid were present in all subjects until 4 h, and5 of them were positive at 6 h post-administration. Oral fluid, in contrast to plasma, is a suitable, non-invasive, and easy biological matrix to collect in a non-controlled setting. Nevertheless, the interpretation of oral fluid 2C-E concentrations without data from plasma is extremely difficult (not obtained in this study or any other).

Our study has several limitations mainly associated with its design as naturalistic-observational. An expectancy bias could appear due to the non-placebo-controlled design. Because participants selected the dose according to their preferences, it resulted in low-moderate doses (ranging from 6.5 to 25 mg), and some doses were only used by one participant. A limited number of subjects could be responsible for a lack of power in some measures. Our findings may not refer to other 2C-E routes of administration. Moreover, the recreational setting could have influenced the effects reported by participants. The limited number of time-point measures did not permit to know the real peak effect/concentration times that will need more intensive evaluations. However, it should be noted that there are a number of strengths: the participation of female subjects, the dose selection by the subjects according to their preferences (6.5–25 mg representing real-life quantities), effects previously experienced with the same or similar psychedelic substances, the recreational scenario, and the use of validated rating scales, questionnaires, and analytic techniques. We cannot discard that a more controlled dose-response study using defined drug doses equal for all subjects would produce a different picture. Future studies should be carried out in controlled conditions and with a larger sample. In addition, it should be noted that 2C-E profiles may vary considerably due to the dose administered and the interindividual differences in pharmacodynamic-pharmacokinetics.

## Conclusion

The results of this non-controlled, observational study in a real-life setting of recreational use provide useful preliminary data of the acute pharmacodynamic effects and pharmacokinetics in oral fluid of 2C-E. Taken together, the current findings suggest that self-administered oral 2C-E induced a constellation of alterations in perceptions, hallucinations, and euphoric-mood which displayed marked similarities to psychedelic experience. Even at low-moderate doses, notable perceptual changes and hallucinations were the most prominent 2C-E effects. High interindividual variability among doses was observed. Participants with self-administered higher doses were more susceptible to experiencing the most intense subjective effects. Based on these preliminary data, oral fluid can be an appropriate, non-invasive, biologic matrix to detect acute 2C-E use.

It can be concluded that further research in humans is needed to compare the effects of 2C-E with other classical and new psychedelic substances.

## Data Availability Statement

The datasets generated for this study are available on request to the senior author (MF, magi.farre@uab.cat).

## Ethics Statement

The protocol was approved by the local Research Ethics Committee (CEIC-Parc de Salut Mar, Barcelona, Spain). The study was conducted in accordance with the Declaration of Helsinki and Spanish laws concerning clinical research. The participants provided their written informed consent previous to participate in this study.

## Author Contributions

MF, RT, MV, MG, MT, ES, JR, and EO conceptualized the study design. MF, EO, MG, ES, and MV collected the data. EO and OP analyzed the oral fluid. MV analyzed the 2C-E contents. EP and CP-M analyzed the data. EP, EO, CP-M, MT, MG, MV, OP, ES, JR, RT, and MF wrote, revised, and approved the manuscript.

## Conflict of Interest

The authors declare that the research was conducted in the absence of any commercial or financial relationships that could be construed as a potential conflict of interest. The reviewer ML declared a past co-authorship with one of the authors JR to the handling Editor.

## Footnotes

1 https://www.globaldrugsurvey.com/

2 https://www.erowid.org/

## Supplementary Material

FIGURE S1 Time course of individual changes from baseline for selected physiological effects (*n* = 10; mean, standard error).

FIGURE S2 Time course of individual changes from baseline for selected subjective effects (*n* = 10; mean, standard error).

FIGURE S3 Time course of individual 2C-E concentrations in oral fluid (*n* = 10; mean, standard error).

## References

[B1] Aarde S. M. Taffe M. A. (2017). Predicting the abuse liability of entactogen-class, new and emerging psychoactive substances via preclinical models of drug self-administration. *Curr. Top. Behav. Neurosci.* 32 145–164. 10.1007/7854_2016_54 27909988

[B2] Bouso J. C. Pedrero-Pérez E. J. Gandy S. Alcázar-Córcoles M. Á (2016). Measuring the subjective: revisiting the psychometric properties of three rating scales that assess the acute effects of hallucinogens. *Hum. Psychopharmacol.* 31 356–372. 10.1002/hup.2545 27470427

[B3] Bruno R. Matthews A. J. Dunn M. Alati R. McIlwraith F. Hickey S. (2012). Emerging psychoactive substance use among regular ecstasy users in Australia. *Drug Alcohol. Depend.* 124 19–25. 10.1016/j.drugalcdep.2011.11.020 22209387

[B4] Cami J. Farré M. Mas M. Roset P. N. Poudevida S. Mas A. (2000). ). Human pharmacology of 3,4-methylenedioxymethamphetamine (“ecstasy”): psychomotor performance and subjective effects. *J. Clin. Psychopharmacol.* 20 455–466. 10917407 10.1097/00004714-200008000-00010

[B5] Caudevilla-Gálligo F. Riba J. Ventura M. González D. Farré M. Barbanoj M. J. (2012). 4-Bromo-2,5-dimethoxyphenethylamine (2C-B): presence in the recreational drug market in Spain, pattern of use and subjective effects. *J. Psychopharmacol.* 26 1026–1035. 10.1177/0269881111431752 22234927

[B6] Dean B. V. Stellpflug S. J. Burnett A. M. Engebretsen K. M. (2013). 2C or not 2C: phenethylamine designer drug review. *J. Med. Toxicol.* 9 172–178. 10.1007/s13181-013-0295-x 23494844 PMC3657019

[B7] Dolder P. C. Schmid Y. Steuer A. E. Kraemer T. Rentsch K. M. Hammann (2017). Pharmacokinetics and pharmacodynamics of lysergic acid diethylamide in healthy subjects. *Clin. Pharmacokinet* 56 1219–1230. 10.1007/s40262-017-0513-9 28197931 PMC5591798

[B8] Eshleman A. J. Forster M. J. Wolfrum K. M. Johnson R. A. Janowsky A. Gatch M. B. (2014). Behavioral and neurochemical pharmacology of six psychoactive substituted phenethylamines: mouse locomotion, rat drug discrimination and in vitro receptor and transporter binding and function. *Psychopharmacology* 231 875–888. 10.1007/s00213-013-3303-6 24142203 PMC3945162

[B9] Farré M. Tomillero A. Pérez-Mañá C. Yubero S. Papaseit E. Roset P. N. (2015). Human pharmacology of 3,4-methylenedioxymethamphetamine (MDMA, ecstasy) after repeated doses taken 4 h apart Human pharmacology of MDMA after repeated doses taken 4 h apart. *Eur. Neuropsychopharmacol.* 25 1637–1649. 10.1016/j.euroneuro.2015.05.007 26073279

[B10] Global Drug Survey [GDS] (2018). *Global Drug Survey [GDS].* Available online at: https://www.globaldrugsurvey.com/gds-2018/ (accessed August 10, 2019).

[B11] González D. Torrens M. Farré M. (2015). Acute effects of the novel psychoactive drug 2C-B on emotions. *Biomed. Res. Int.* 2015:643878. 10.1155/2015/643878 26543863 PMC4620274

[B12] González D. Ventura M. Caudevilla F. Torrens M. Farre M. (2013). Consumption of new psychoactive substances in a Spanish sample of research chemical users. *Hum. Psychopharmacol.* 28 332–340. 10.1002/hup.2323 23881881

[B13] Griffiths R. R. Richards W. A. McCann U. Jesse R. (2006). Psilocybin can occasion mystical-type experiences having substantial and sustained personal meaning and spiritual significance. *Psychopharmacology* 187 268–283. 10.1007/s00213-006-0457-5 16826400

[B14] Iwersen-Bergmann S. Lehmann S. Heinemann A. Schröder C. Müller A. Jungen H. (2019). Masspoisoning with NPS: 2C-E and bromo-dragon fly. *Int. J. Legal Med.* 133 123–129. 10.1007/s00414-018-1882-9 29959557

[B15] Johnson M. W. MacLean K. A. Reissig C. J. Prisinzano T. E. Griffiths R. R. (2011). Human psychopharmacology and dose-effects of salvinorin A, a kappa opioid agonist hallucinogen present in the plant Salvia divinorum. *Drug Alcohol. Depend.* 115 150–155. 10.1016/j.drugalcdep.2010.11.005 21131142 PMC3089685

[B16] Lamas X. Farré M. Llorente M. Camí J. (1994). Spanish version of the 49-item short form of the Addiction Research Center Inventory (ARCI). *Drug Alcohol. Depend.* 35 203–209. 10.1016/0376-8716(94)90075-2 7956749

[B17] Martínez-Riera R. Pérez-Mañá C. Papaseit E. Fonseca F. de la Torre R. Pizarro N. (2019). Soy Isoflavone extract does not increase the intoxicating effects of acute alcohol ingestion in human volunteers. *Front. Pharmacol.* 10:131 10.3389/fphar.2019.00131 PMC6400998 30873023

[B18] Mas M. Farré M. de la Torre R. Roset P. N. Ortuño J. Segura J. (1999). Cardiovascular and neuroendocrine effects and pharmacokinetics of 3,4-methylenedioxymethamphetamine in humans. *J. Pharmacol. Exp. Ther.* 290 136–145. 10381769

[B19] Matthews A. Sutherland R. Peacock A. Van Buskirk J. Whittaker E. Burns L. (2016). I like the old stuff better than the new stuff– Subjective experiences of new psychoactive substances. *Int. J. Drug Policy* 40 44–49. 10.1016/j.drugpo.2016.11.004 27939599

[B20] Nagai F. Nonaka R. Satoh Hisashi Kamimura K. (2007). The effects of non-medically used psychoactive drugs on monoamine neurotransmission in rat brain. *Eur. J. Pharmacol.* 22 132–137. 17223101 10.1016/j.ejphar.2006.11.075

[B21] Nichols D. E. (2016). Psychedelics. *Pharmacol. Rev.* 68 264–355. 10.1124/pr.115.011478 26841800 PMC4813425

[B22] Observatorio de Drogas de Colombia [ODC] (2017). *Reporte de Drogas de Colombia.* Available online at: http://www.odc.gov.co/Portals/1/publicaciones/pdf/odc-libro-blanco/reporte_drogas_colombia_2017.pdf (accessed August 10, 2019).

[B23] Palamar J. J. Barratt M. J. Ferris J. A. Winstock A. R. (2016). Correlates of new psychoactive substance use among a self-selected sample of nightclub attendees in the United States. *Am. J. Addict.* 25 400–407. 10.1111/ajad.12403 27419383 PMC5072356

[B24] Papaseit E. Farré M. Pérez-Mañá C. Torrens M. Ventura M. Pujadas M. (2018). Acute pharmacological effects of 2C-B in humans: an observational study. *Front. Pharmacol.* 9:206. 10.3389/fphar.2018.00206 29593537 PMC5859368

[B25] Papaseit E. Pérez-Mañá C. Mateus J. A. Pujadas M. Fonseca F. Torrens M. (2016). Human pharmacology of mephedrone in comparison with MDMA. *Neuropsychopharmacology* 41 2704–2713. 10.1038/npp.2016.75 27206266 PMC5026738

[B26] Polito V. Stevenson R. J. (2019). A systematic study of microdosing psychedelics. *PLoS One* 14:e0211023. 10.1371/journal.pone.0211023 30726251 PMC6364961

[B27] Puxty D. J. Ramaekers J. G. de la Torre R. Farré M. Pizarro N. Pujadas M. (2017). MDMA-Induced dissociative state not mediated by the 5-HT(2A) receptor. *Front. Pharmacol.* 8:455. 10.3389/fphar.2017.00455 28744219 PMC5504523

[B28] Riba J. Anderer P. Jané F. Saletu B. Barbanoj M. J. (2004). Effects of the South American psychoactive beverage ayahuasca on regional brain electrical activity in humans: a functional neuroimaging study using low-resolution electromagnetic tomography. *Neuropsychobiology* 50 89–101. 10.1159/000077946 15179026

[B29] Riba J. Rodríguez-Fornells A. Urbano G. Morte A. Antonijoan R. Montero M. (2001). Subjective effects and tolerability of the South American psychoactive beverage Ayahuasca in healthy volunteers. *Psychopharmacology* 154 85–95. 10.1007/s002130000606 11292011

[B30] Rickli A. Luethi D. Reinisch J. Buchy D. Hoener M. C. Liechti M. E. (2015). Receptor interaction profiles of novel N-2-methoxybenzyl (NBOMe) derivatives of 2,5-dimethoxy-substituted phenethylamines (C drugs). *Neuropharmacology* 99 546–553. 10.1016/j.neuropharm.2015.08.034 26318099

[B31] Sacks J. Ray M. J. Williams S. Opatowsky M. J. (2012). Fatal toxic leukoencephalopathysecondary to overdose of a new psychoactive designer drug 2C-E (”Europa”). *Proc* 25 374–376. 23077393 10.1080/08998280.2012.11928883 PMC3448584

[B32] Schmid Y. Enzler F. Gasser P. Grouzmann E. Preller K. H. Vollenweider F. X. (2015). Acute effects of lysergic acid diethylamide in healthy subjects. *Biol. Psychiatry* 78 544–553. 10.1016/j.biopsych.2014.11.015 25575620

[B33] Sexton J. D. Crawford M. S. Sweat N. W. Varley A. Green E. E. Hendricks P. S. (2019). Prevalence and epidemiological associates of novel psychedelic use in the United States adult population. *J. Psychopharmacol.* 28:269881119827796. 10.1177/0269881119827796 30816808

[B34] Shulgin A. Shulgin A. (1990). *PIHKAL: A Chemical Love Story.* Berkeley, CA: Transform Press.

[B35] Sutherland R. Bruno R. Peacock A. Lenton S. Matthews A. Salom C. (2017). Motivations for new psychoactive substance use among regular psychostimulant users in Australia. *Int. J. Drug Policy* 43 23–32. 10.1016/j.drugpo.2016.12.021 28161577

[B36] Sutherland R. Peacock A. Whittaker E. Roxburgh A. Lenton S. Matthews A. (2016). New psychoactive substance use among regular psychostimulant users in Australia, 2010-2015. *Drug Alcohol. Depend* 161 110–118. 10.1016/j.drugalcdep.2016.01.024 26880592

[B37] Theobald D. S. Fritschi G. Maurer H. H. (2007). Studies on the toxicological detection of the designer drug 4-bromo-2,5-dimethoxy-beta-phenethylamine (2C-B) in rat urine using gas chromatography-mass spectrometry. *J. Chromatogr. B Analyt. Technol. Biomed. Life Sci.* 846 374–377. 10.1016/j.jchromb.2006.08.049 16978931

[B38] Topeff J. Ellsworth H. Willhite L. Bangh S. Edwards E. M. Cole J. (2011). A case series of symptomatic patients, including one fatality, following 2C-E exposure. *Clin.Toxicol.* 49:526.

[B39] Tracy D. K. Wood D. M. Baumeister D. (2017). Novel psychoactive substances: types, mechanisms of action, and effects. *BMJ* 356:i6848. 10.1136/bmj.i6848 28122697

[B40] United Nations Office on Drugs and Crime [UNODC] (2014). *Global Synthetic Drugs Assessment Amphetamine-type stimulants and New Psychoactive Substances.* Available online at: https://www.unodc.org/documents/scientific/2014_Global_Synthetic_Drugs_Assessment_web.pdf (accessed August 10, 2019).

[B41] Van Vrancken M. J. Benavides R. Wians F. H. Jr. (2013). Identification of designer drug2C-E (4-ethyl-2, 5-dimethoxy-phenethylamine) in urine following a drug overdose. *Proc* 26 58–61. 23382618 10.1080/08998280.2013.11928922 PMC3523774

[B42] Vollenweider F. X. (2001). Brain mechanisms of hallucinogens and entactogens. *Dial. Clin. eurosci.* 3 265–279. 10.31887/DCNS.2001.3.4/fxvollenweider PMC3181663 22033605

