# Supplementary figures and images for: Acute Effects of 2C-E in Humans: An Observational Study
Source: Front Pharmacol. 2020 Mar 18;11:233. doi: 10.3389/fphar.2020.00233 (PMC7093582; doi:10.3389/fphar.2020.00233)

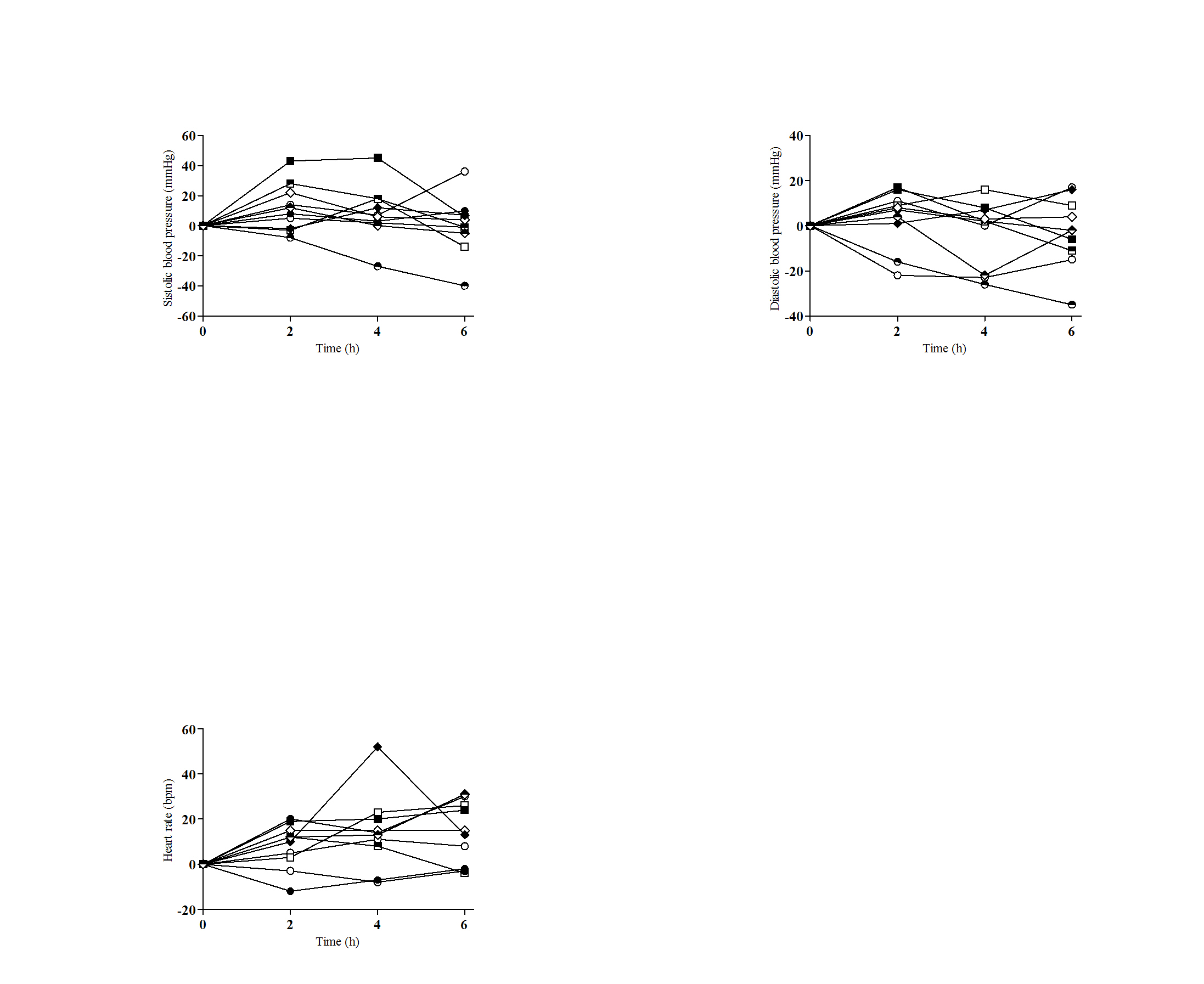

Supplement: FIGURE S1 — Time course of individual changes from baseline for selected physiological effects (n = 10; mean, standard error). [file Image_1.JPEG]

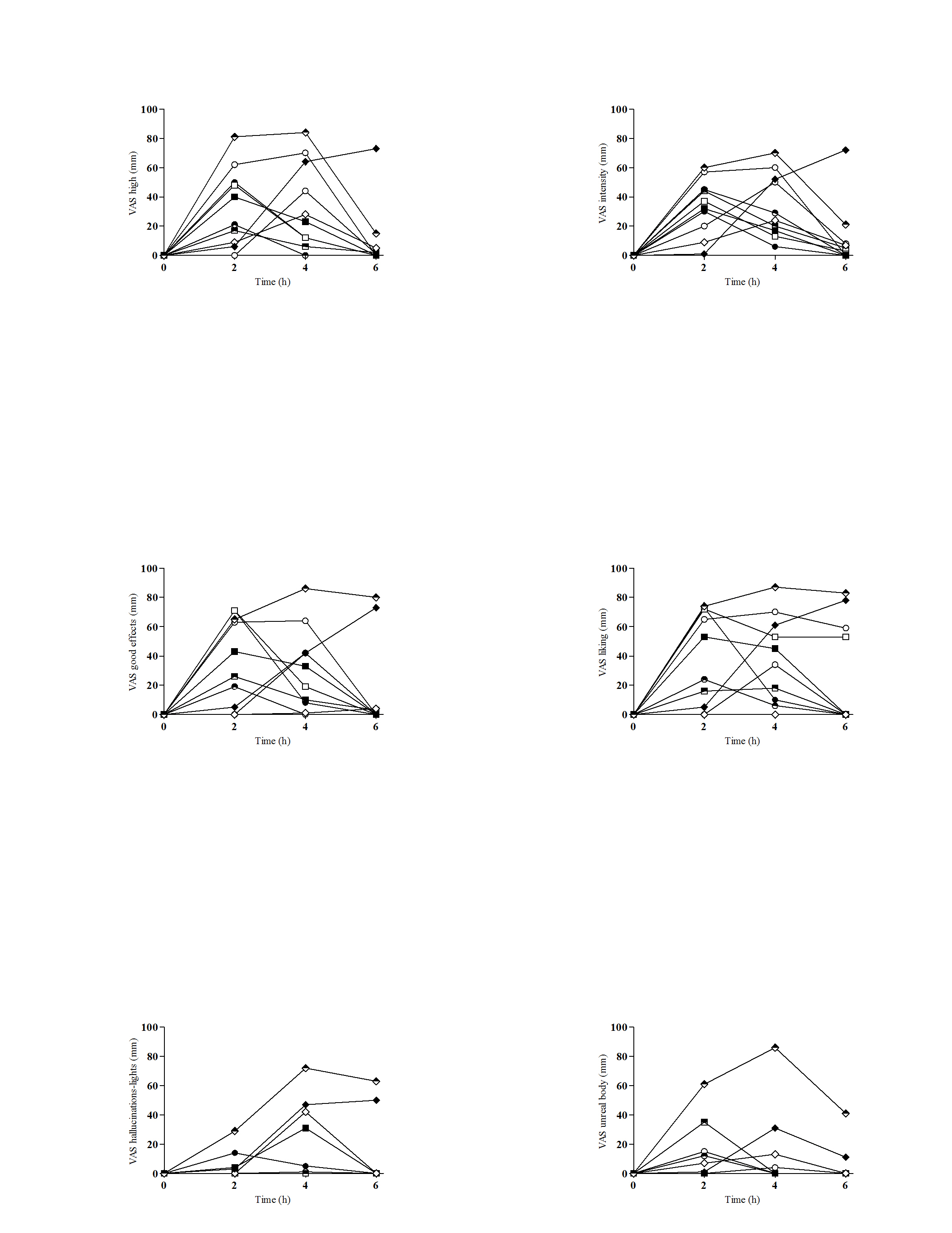

Supplement: FIGURE S2 — Time course of individual changes from baseline for selected subjective effects (n = 10; mean, standard error). [file Image_2.JPEG]

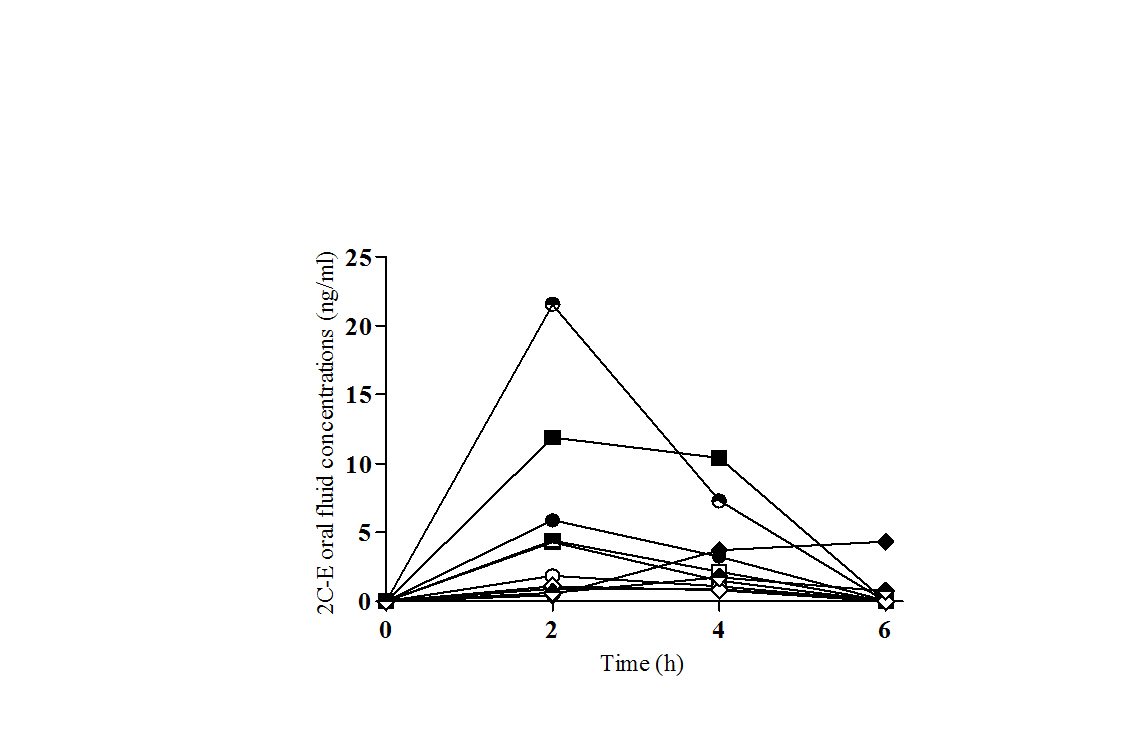

Supplement: FIGURE S3 — Time course of individual 2C-E concentrations in oral fluid (n = 10; mean, standard error). [file Image_3.JPEG]
